# Effects of obstructive sleep apnea on non-alcoholic fatty liver disease in patients with obesity: a systematic review

**DOI:** 10.1038/s41366-023-01378-2

**Published:** 2023-09-11

**Authors:** Mohamed Hany, Anwar Ashraf Abouelnasr, Mohamed Hesham Abdelkhalek, Mohamed Ibrahim, Mostafa R. Aboelsoud, Adel Ibrahim Hozien, Bart Torensma

**Affiliations:** 1 Department of Surgery, Medical Research Institute, Alexandria University, Alexandria Governorate, Egypt; 2 Madina Women’s Hospital (IFSO certified center, European chapter), Alexandria Governorate, Egypt; 3 Department of Anesthesia and pain management, Medical Research Institute Alexandria University, Alexandria Governorate, Egypt; 4 Leiden University Medical Center (LUMC), Leiden, The Netherlands

**Keywords:** Obesity, Epidemiology

## Abstract

**Introduction:**

Obesity has been linked to non-alcoholic fatty liver disease (NAFLD), a widespread chronic liver ailment, as well as obstructive sleep apnea (OSA). The development of NAFLD is influenced by repeated intermittent hypoxia, a feature of OSA.

**Methods:**

This systematic review (SR) investigated CENTRAL, PubMed, and EMBASE databases. The endpoint of this SR was to assess which OSA-related indicators could predict the presence of NAFLD and the effect of bariatric metabolic surgery (BMS) on improving OSA and NAFLD over time.

**Results:**

Compared to previous SRs published in 2013, 14 new publications were added to our SR, alongside studies conducted prior to 2013. The SR ultimately included 28 studies (18 cross-sectional and 10 cohort trials). In the majority of studies, significant correlations were observed between OSA, OSA-related outcomes, and NAFLD. However, the apnea-hypopnea index (AHI) alone proved to be an inadequate predictor of NAFLD. Instead, respiratory and metabolic changes were found to alleviate oxidative stress induced by hypoxemia.

Six studies involved patients who underwent BMS, with one evaluating patients before and after BMS, revealing associations between increased OSA and NAFLD improvement following BMS. Six months after surgery, 100% of patients in the mild-to-moderate OSA group were free from fatty liver, and an 89% reduction was observed in the severe OSA group.

**Conclusion:**

For the first time, BMS has been tested in treating both OSA and NAFLD pre and postoperative with positive results. Further research, ideally with histological and functional data, is needed to confirm these findings. The SR identified 14 distinct liver outcome tests; however, high heterogeneity and incomplete data precluded a meta-analysis. It is imperative to pay greater attention to the influence of OSA-related factors and uniformity in liver outcomes testing concerning NAFLD. To accomplish this, study designs should be enhanced by incorporating more comprehensive pre- and postoperative evaluations, extending follow-up periods, and employing a more consistent methodology for liver diagnosis in patients with obesity.

## Introduction

Obesity, as in patients with a BMI ≥ 35, is associated with severe medical problems, including non-alcoholic fatty liver disease (NAFLD), considered one of the most currently prevalent chronic liver diseases [1]. NAFLD can be divided into four stages that go from simple fatty liver (steatosis), which is stage 1, to stage 2, which involves non-alcoholic steatohepatitis (NASH) and more inflammation; stage 3 fibrosis, where persistent inflammation causes scar tissue around the liver, and finally stage 4, cirrhosis, which is the most severe stage. After years of inflammation, the liver eventually shrinks and becomes scarred and lumpy causing permanent damage and even liver failure [1] and since the prevalence of NAFLD in patients with obesity is approximately 60–80%, adequate treatment is necessary [2]

The need for a new systematic review at this juncture arises from several limitations in previous works. In 2013, two significant systematic reviews were undertaken to explore the relationship between OSA and NAFLD in patients with obesity [3, 4]. Notably, despite using identical search strategies, these reviews included different studies, potentially leading to a fragmented understanding of the association. Additionally, both reviews encompassed adults with overweight and children in their analysis, introducing a potential bias toward the results specific to patients with obesity. Furthermore, neither review considered the impact of follow-up periods post-weight loss treatment, leaving this crucial aspect unexplored.

Bariatric metabolic surgery (BMS) has undergone rigorous investigation as a treatment for NAFLD over the past decade, with a substantial body of evidence emerging from systematic reviews (SR) [5–11]. For instance, an SR conducted in 2022 demonstrated that BMS’s effectiveness in reducing the resolution of steatosis was improved in 56% of patients, ballooning degeneration in 49%, inflammation in 45%, and fibrosis in 25% [10]. This reflects the generally favorable outcomes associated with BMS in managing NAFLD. Another factor linked to NAFLD is obstructive sleep apnea (OSA). OSA has a prevalence of 4–24% in men and 2–9% in women without obesity but the prevalence increases to 30–50% in patients with obesity [12]. OSA is characterized by recurrent upper airway collapse during sleep, resulting in sleep fragmentation and recurrent oxyhemoglobin desaturation, termed chronic intermittent hypoxia [12]. Repeated intermittent hypoxia, one of the characteristics of OSA, is related to the development of NAFLD [13]. Hypoxia, a condition in which the liver is deprived of adequate oxygen supply, contributes to the pathogenesis of NAFLD. This susceptibility to hypoxia has led to the proposition of a ‘two-hit’ hypothesis to explain the disease process. The ‘first hit’ refers to the accumulation of excess hepatic triglycerides, primarily driven by dysregulation of fatty acids and insulin resistance. Importantly, this occurs without significant alcohol consumption or other liver diseases. The ‘second hit’ is induced by hypoxia and other triggers, resulting in an increase in oxidative stress and cytokine expression. This escalates the simple steatosis (stage 1) to a more severe form, steatohepatitis (stage 2), thereby progressing the disease [14].

Liver biopsy remains the gold standard for diagnosing the stages of NAFLD. It allows for determining the severity of NAFLD based on the NAFLD activity score (NAS). This scoring system assesses the degree of steatosis, lobular inflammation, hepatocellular ballooning, and fibrosis [15] Fibrosis is not included as a component of NAS because it is less reversible and does not reflect the severity of the inflammatory process. NAS ≥ 5 is considered to indicate non-alcoholic steatohepatitis (NASH), whereas NAS < 3 excludes the presence of NASH [15]. Additionally, serum alanine and aspartate aminotransferases (ALT and AST) indicate liver injury but are neither sensitive nor specific enough for diagnosing NAFLD nor characterizing its severity [15].

Our systematic review aims to address these limitations by assessing which OSA-related indicators can predict the presence of NAFLD and the effect of bariatric metabolic surgery (BMS) on improving OSA and NAFLD over time.

## Methods

This systematic review (SR) was conducted in accordance with the PRISMA Reporting Items for Systematic Reviews and Meta-Analyses (PRISMA) guidelines by Moher et al. [16]. (Checklist appendix 1). This SR was registered at PROSPERO (protocol ID CRD42022361882).

### Search strategy

The Cochrane Central Register of Controlled Trials (CENTRAL), PubMed, and EMBASE were searched from their inception until October 8th^,^ 2022. We used the following terms and their synonyms, truncated where necessary: *obstructive sleep apnea, hypoxia, desaturation, nonalcoholic fatty liver disease, apnea-hypopnea-index, obesity, bariatric surgery, or roux en y gastric bypass, or sleeve gastrectomy*. Grey literature was also searched, and a reference crosscheck was performed to identify eligible articles not identified through previous searches. The search was conducted without restrictions on language or publication date.

## Study eligibility criteria

### Type of studies

#### Included

Randomized controlled trials (RCTs), prospective and retrospective cohort studies, cross-sectional studies (CS), and case-control studies were included. RCTs and non-randomized studies directly compared the outcomes of interest.

#### Excluded

Descriptive studies, case series, and case reports were excluded because of their reduced level of evidence.

#### Endpoints

The endpoint of this SR was to assess which OSA-related indicators could predict the presence of NAFLD and the effect of bariatric metabolic surgery (BMS) on improving OSA and NAFLD over time.

### Types of participants

Inclusion criteria for participants were: patients aged 18 years or older; BMI of 40 kg/m^2^ or BMI 35 kg/m^2^ with associated medical problems; non-NAFLD or NAFLD and the presence of OSA.

### Type of liver diagnoses

The literature was searched for all diagnostic variables used to detect NAFLD and correlated with the presence of OSA in patients with obesity.

### Study selection and data extraction

The titles and abstracts of the studies were screened by three independent reviewers (BT, MHi, AE) based on the inclusion and exclusion criteria in the Covidence systematic review software (Veritas Health Innovation, Melbourne, Australia). Subsequently, the same reviewers independently reviewed the remaining full-text reports for eligibility. Data from the full-text articles were extracted independently. In all stages, disagreements were resolved by discussion or consulting a fourth independent reviewer (MHa). Data on the outcomes were collected and divided into separate groups for analysis. The studies were stratified based on the diagnostic tools for NAFLD.

### Assessment of risk of bias

Three reviewers (BT, MH, and AE) independently assessed the risk of bias for the methodological quality of each included study using the Newcastle-Ottawa quality assessment scale for cohort studies, which is divided into three domains (selection bias, comparability, and outcome bias) what includes eight questions. The maximum score is nine points [17]. The JBI critical appraisal tool for cross-sectional studies was used (selection, exposure, confounding, measurements, and statistical approach in eight questions), with a maximum score of eight points [18]. GRADE assessment of the score was applied to all included studies [19].

### Statistical analysis

All the studies were extracted from Jotform Inc. (4 Embarcadero Center, Suite 780, San Francisco, CA 94111) and classified as either cross-sectional or cohort study design. The characteristics of liver variables and OSA are displayed according to the definitions used in the articles. A meta-analysis was conducted if appropriate. Heterogeneity was evaluated using the tau-square (I [2]) statistic. I^2^ of 0–40% was considered as low heterogeneity, 30–50% as moderate heterogeneity, 50–75% as substantial heterogeneity, and 75–100% as high heterogeneity, respectively. When heterogeneity was greater than 60% or if definitions of the outcome for OSA or liver were not possible, or outcomes were not displayed, a meta-analysis was not used, and the outcomes of the articles were described in the SR. Statistical significance was set at *P* < 0.05. All analyses were performed using Review Manager version 5.4.1.

## Results

### Literature search

**Fig. 1:**
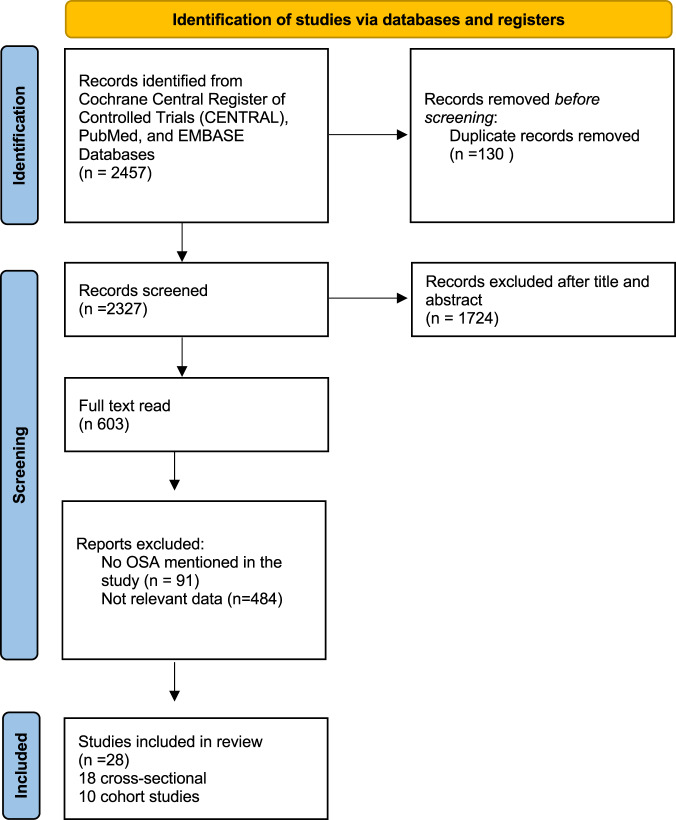
PRISMA flow diagram. PRISMA 2020 flow diagram for OSA and NAFLD.

In total, 2457 references were imported for screening, and 130 duplicates were removed. Of the 2327 remaining studies, 1724 were excluded after screening their titles and abstracts. After evaluating 603 full-text articles, this systematic review included in total 28 studies. (18 cross-sectional studies [20–37], and 10 cohort trials [38–47]) (Fig. 1).

### Characteristics of the included studies

From 2007 to 2022, 18 cross-sectional studies were conducted globally, with a median of 106 patients per study. Ten cohort trials from 2007 to 2021 were split into five retrospectives and five prospective studies, with a median of 123 patients per study. Six cross-sectional and five cohort studies involved non-BMS patients with obesity, OSA, and NAFLD. Ten cross-sectional and one cohort study included BMS patients, while three cross-sectional and three cohort studies focused on post-BMS patients. (Table 1).

**Table 1:** Baseline study characteristics cross-sectional and cohort studies.

Cross-sectional studies								
Author	Year	Country	Total sample size	NAFLD diagnosed	OSAS diagnosed	CPAP use	Bariatric metabolic surgery	Conclusion
Jouët et al.	2007	France	62	Biopsy/histology AST and ALT elevation	PSG with AHI and Spo2	No CPAP use	Opt-in/ planning for BMS	The study did not find a relationship between OSA and NASH or other liver lesions in patients with severe obesity
Mishra et al.	2008	U.S.A.	101	Biopsy/histology	PSG with AHI and Spo2	Uses CPAP	Opt-in/ planning for BMS	Frequent nocturnal hypoxic episodes in patients with severe obesity and OSA may be a risk factor for developing steatohepatitis and fibrosis
Campos et al.	2008	U.S.A.	200	Biopsy/histology AST and ALT elevation	PSG with AHI	Uses CPAP	Opt-in/ planning for BMS	The proposed clinical scoring can predict NAFLD in patients with severe obesity and OSA, with a present OR of 4.0 for OSA.
Daltro et al.	2010	Brazil	40	Biopsy/histology	PSG with AHI and Spo2	No CPAP use	Opt-in/ planning for BMS	This study showed that patients with obesity had elevated OSA and NAFLD frequencies.
Turkay et al.	2011	Turkey	106	Radiology	PSG with AHI and Spo2	N.A./ N.M.	No BMS	The possibility raise that OSA may be a novel risk factor for NAFLD when patients have Repetitive oxyhaemoglobin desaturation or severe hypoxemia inflammation and oxidative stress. treatment of OSA may delay the development or reduce the severity of NAFLD.
Aron-Wisnewsky et al.	2012	France	101	Biopsy/histology	N.A./N.M.	No CPAP use	Had BMS	Chronic intermittent hypoxia with the highest ODI was strongly associated with more severe liver injuries in patients with severe obesity
Corey et al.	2013	U.S.A.	159	Biopsy/histology	Known OSA from history files	Uses CPAP	Had BMS	Absence of OSA protect against NAFLD in patients with obesity.
Mesarwi et al.	2015	U.S.A.	35	Biopsy/histology	PSG with AHI and Spo2	Uses CPAP	Had BMS	Hepatic fibrosis are related to the severity of OSA in patients with severe obesity
Benotti et al.	2016	U.S.A.	362	Radiology AST and ALT elevation	PSG with AHI	No CPAP use	Opt-in/ planning for BMS	OSA severity and its accompanying hypoxia are associated with the severity of NAFLD in patients with obesity
Scartabelli et al.	2018	Australia	97	Radiology AST and ALT elevation	PSG with AHI and Spo2	N.A./ N.M.	No BMS	hepatic left lobe volume is predicting the presence of OSA in patients with obesity
Bhatt et al.	2019	India	171	Radiology	PSG with AHI	N.A./ N.M.	No BMS	Systemic inflammation is more pronounced in Asian Indians patients with overweight/obesity, OSA and NAFLD
Zhang, L et al.	2020	China	153	Biopsy/histology Radiology	PSG with AHI and Spo2	N.A./ N.M.	Opt-in/ planning for BMS	Confirmed that OSA may play a role in liver injury in a population with severely obesity with NAFLD
Schwenger et al.	2020	Canada	61	Biopsy/histology	PSG with AHI and Spo2	N.A./ N.M.	Opt-in/ planning for BMS	OSA parameters (AHI and lower minimum oxygen saturation) were significantly associated with the presence of NAFLD and lobular inflammation.
Tomar et al.	2021	India	78	Biopsy/histology Radiology	PSG with AHI and Spo2	N.A./ N.M.	No BMS	NAFLD patients have a higher prevalence and greater severity of OSA OSA is independent of obesity among patients with chronic liver disease but prevalent among the NAFLD group.
Kim, T. et al.	2022	South Korea	4275	AST and ALT elevation	N.A./ N.M.	N.A./ N.M.	No BMS	OSA is closely associated with NAFLD in the general population. prevalence of NAFLD were more accentuated in people with obesity
Grillo et al.	2022	Brazil	155	Biopsy/histology	PSG with AHI	N.A./ N.M.	No BMS	Prevalence of OSAS was high in the patients with NAFLD and was independently associated with BMI
Fu et al.	2022	China	183	Biopsy/histology	PSG with AHI and Spo2	No CPAP use	Opt-in/ planning for BMS	Chronic intermittent hypoxia caused by OSA may aggravate NAFLD and lead to a higher risk of NAFLD in patients with obesity
Bettini et al.	2022	Italy	334	Biopsy/histology	PSG with AHI and Spo2	No CPAP use	Opt-in/ planning for BMS	Presence and severity of the OSA in NAFLD was found. Also an association of fibrosis, and OSA severity in adult patients with severe obesity

Prospective and retrospective studies									
Author	Year	Study design	Country	Total sample size	NAFLD diagnosed	OSAS diagnosed	CPAP use	Bariatric metabolic surgery	Conclusion
Kallwitz et al.	2007	Retrospective cohort	U.S.A.	85	Biopsy/histology	PSG with AHI	N.A./ N.M.	Opt-in/ planning for BMS	In patients with obesity with NAFLD, OSA was associated with elevated alanine aminotransferase levels and a trend toward histologic evidence of progressive liver disease
Acarturk et al.	2007	Prospective cohort	Turkey	45	Radiology AST and ALT elevation	PSG with AHI	No CPAP use	No BMS	There was no correlation between the severity of liver steatosis and OSA or AHI/ODI
Polotsky et al.	2009	Prospective cohort	U.S.A.	90	Biopsy/histology AST and ALT elevation	Respiratory disturbance index	No CPAP use	Opt-in/ planning for BMS	Hypoxic stress of OSA predicts the severity of insulin resistance and may impact on the progression of NAFLD in patients with severe obesity
Ulitsky et al.	2010	Retrospective cohort	U.S.A.	253	Biopsy/histology	PSG with AHI and Spo2	N.A./N.M.	Had BMS	a simple scoring system which incorporates diabetes, OSA, ALT, performs well in predicting NAFLD
Byrne et al.	2012	Retrospective cohort	U.S.A.	53	AST and ALT elevation	PSG with AHI	No CPAP use	No BMS	OSA is an independent risk factor for liver injury/NAFLD
Weingarten et al.	2012	Retrospective cohort	U.S.A.	218	Biopsy/histology	PSG with AHI and Spo2	Uses CPAP	Had BMS	Patients with severe obesity evaluated before bariatric surgery and a severity of OSA were not associated with NAFLD severity
Agrawal et al.	2015	Prospective cohort	India	123	Radiology AST and ALT elevation	PSG with AHI	N.A./N.M.	Had BMS	Low prevalence of symptomatic OSA in Indian patients with NAFLD. Occurrence of OSA in patients with NAFLD was predicted by male gender and obesity
Krolow et al.	2020	Prospective cohort	Brazil	51	Biopsy/histology Radiology	PSG with AHI and Spo2	N.A./N.M.	No BMS	The association between NAFLD and moderate to severe OSA, after adjusting for the degree of obesity was present.
Zhang, Y.X et al.	2020	Retrospective cohort	China	308	Biopsy/histology Radiology	PSG with AHI	N.A./N.M.	Had BMS	MBS plays a pivotal role in the control of medical conditions in patients with OSA and NAFLD
Chung et al.	2021	Prospective cohort	South Korea	8.116,525	A surrogate measure of fatty liver	National Health Insurance System	N.A./N.M.	N.A./N.M.	Fatty liver index score may help identify individuals with a high risk of OSA in patients with overweight or obesity.

*N.A.* not applicable, *N.M.* not mentioned, *BMS* Bariatric metabolic surgery, *PSG* Polysomnography, *AHI* apneu hypopnea index.

### Risk of bias

In the cross-sectional studies, the risk of bias was generally low, with only two studies having a high risk due to unclear assessment and confounding factors handling.

**Fig. 2:**
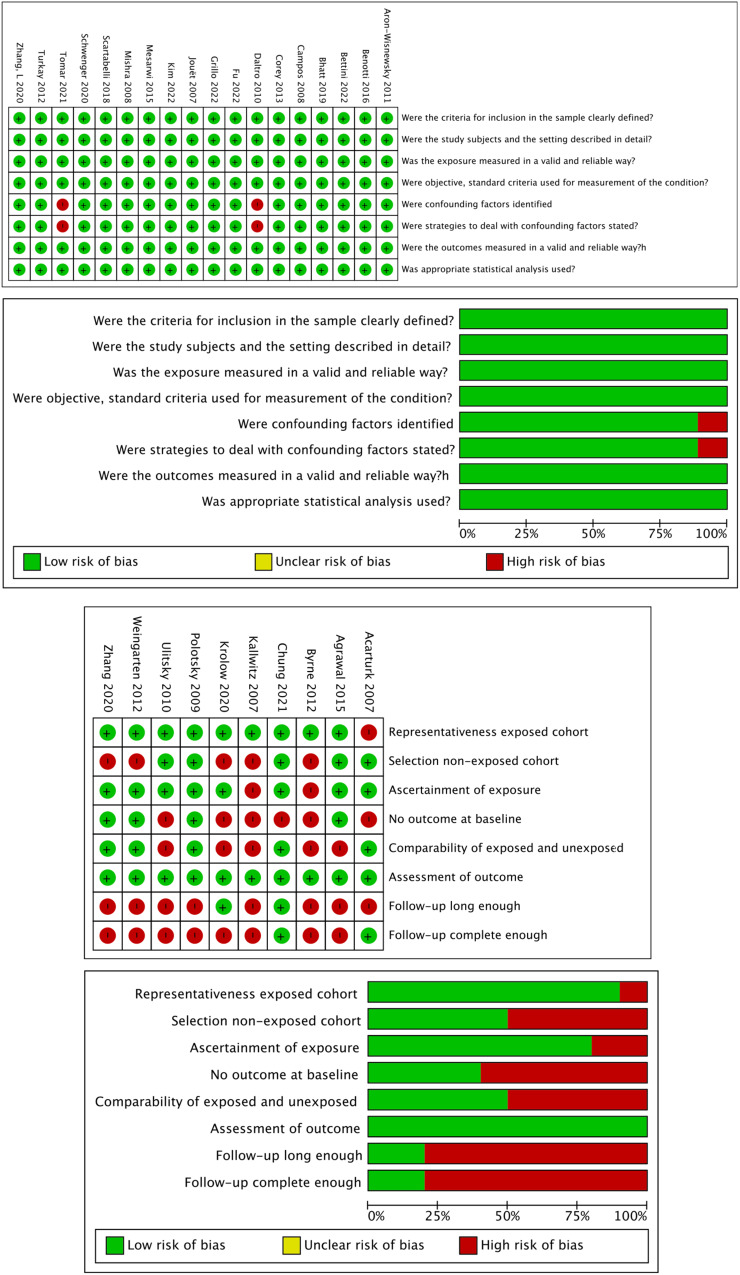
Risk of Bias. **a** Risk of Bias assessment for cross-sectional studies. **b** Risk of Bias assessment for cohort studies.

In the cohort studies, bias risk varied: 10% in exposed cohort representativeness, 50% in non-exposed cohort selection, 20% in exposure ascertainment, 60% in initial outcome absence, 50% in cohort comparability, 0% in outcome assessment, and 80% inadequate follow-up duration. (Fig. 2a, b).

### GRADE assessment

This systematic review showed that all cross-sectional (CS) studies had low-quality grade (good quality). Two studies (Daltro and Tomar et al.) had a medium-quality grade. In the cohort studies, high quality was reported by Byrne and Kallwitz et al. (poor quality), and the rest were of medium quality (fair quality) (Fig. 2a, b).

## Meta-analysis

A meta-analysis was infeasible because of the various variable liver outcome criteria and measurements related to OSA in both cross-sectional and cohort studies, which prevented data pooling. This SR will describe all studies on the outcome and relationship towards the endpoints.

### NAFLD and OSA on BMS

Of 28 studies, 6 involved patients with BMS. Three cross-sectional studies had perioperative liver biopsies without follow-up (Table 1).

Only one cohort study by Zhang et al. (2020) assessed preoperative and 6-month postoperative BMS in OSA and NAFLD patients [44].

In this study, 308 patients underwent BMS, and 181 were diagnosed with OSA. Of these 181 patients, 80 (44%) had mild to moderate OSA, and 101 (55%) had severe OSA. NAFLD was diagnosed in 86% of patients, and moderate and severe NAFLD were more common in the severe OSA group. After six months, sleep apnea and nocturnal hypoxemia were under control (Apnea hypopnea index (AHI) decreased from 18.7 ± 5.9 to 7.7 ± 7.5 in the mild to moderate OSA and from 76.0 ± 32.2 to 22.0 ± 20.4 in severe OSA group). Nevertheless, 23% of patients in this group had severe OSA after six months. A possible explanation for this finding is the short follow-up time.

The liver–spleen ratio (LSR) was calculated pre and postoperatively. A significantly lower LSR was measured in patients with severe OSA and mild-to-moderate OSA (0.68 vs. 0.82). The lower the score, the higher the prevalence of NAFLD. The results showed that 100% of patients in the mild to moderate OSA group were free from fatty liver, while the severe OSA group experienced an 89% reduction in fatty liver (as indicated by a rise in LSR to 1.2 in both OSA groups) after 6 months post-operation.

In addition, advanced liver fibrosis in the severe OSA group resolved 6 months postoperatively in all patients. Finally, a significant improvement in liver function was found. A complete resolution of abnormal liver function was achieved in 94% of the mild-moderate OSA group and 93% of the severe OSA group.

### OSA-related indicators for NAFLD

Described OSA-related outcomes were apnea-hypopnea index (AHI), oxygen desaturation index (ODI), longest apnea time (LAT), mean oxygen saturation (MSO2), nadir oxygen saturation (NSO2), and oxygen saturation less than 90% in total sleep time (SIT90), together with liver parameters, alanine aminotransferase (ALT) and aspartate aminotransferase (AST).

A study by Zhang et al. [44] showed that significant negative correlations were observed preoperatively between AHI, ODI, LAT, SIT90, and LSR (higher OSA-related outcomes led to lower LSR and increased NAFLD presence). Conversely, higher OSA-related outcomes corresponded to increased NFS, ALT, and AST levels, indicating a more significant presence of NAFLD.

Six months postoperatively, significant weight loss occurred (total body weight loss of 24% and 27% in both OSA groups), and significant OSA-related outcomes decreased towards the correlations of the LSR and NFS in the opposite direction as preoperatively.

This study showed, therefore, that OSA is related to NAFLD and, at the same time, that OSA-related outcomes also directly affect NAFLD as a parameter.

### OSA-related indicators on NAFLD in the other included studies

In cross-sectional studies, 15/18 mentioned AHI measurements and 10/18 used SPo2 variables. In cohort studies, 8/10 mentioned AHI measurements and 3/10 used SPo2 variables. In 66% of the cross-sectional studies, a significant OSA-related outcome vs. -NAFLD correlation was found, 14% found no difference, and 20% did not measure this outcome. In cohort studies, 75% did not measure the correlation between OSA-related outcomes vs. NAFLD, 13% found significant differences, and 13% found no differences.

## Cross-sectional studies

Jouët et al. [20] did not observe a correlation between AHI and other OSA-related indicators with NAFLD. Our systematic review sought to provide an explanation for this result, but a clear answer remains elusive. Bettini et al. [21], on the other hand, established a significant correlation between AHI and other OSA-related indicators with the Fibrosis 4 index (FIB-4), a marker predicting NAFLD. Daltro et al. [22] found no significant relationship between AHI and ALT and AST, or other OSA-related indicators for NAFLD, except when AHI exceeded 15, a stronger correlation with NAFLD was noted. Mesarwi et al. [23] and Mishra et al. [24] found that AHI and oxygen desaturation were significantly higher in NAFLD patients and were associated with an increased risk of NAFLD. Campos et al. [25], however, did not analyze the effects of OSA-related outcomes on NAFLD, focusing solely on OSA. Both Tomar et al. [26] and Scartabelli et al. [27] reported significant differences between NAFLD and non-NAFLD patients regarding AHI, oxygen desaturation, and liver volume assessments, which can predict NAFLD. Turkay et al. [30] observed that AHI and ODI values increased in proportion with NAFLD severity. Similarly, Zhang et al. [31] found a significant correlation between AHI and OSA-related outcomes for NAFLD and liver ALT. Grillo et al. [32] only used the AHI parameter to define OSA without testing for other OSA-related indicators of NAFLD. Fu et al. [33] noticed an increase in AHI, ODI, and T90 with the NASH activity grade, indicating that patients with a higher NASH activity grade experienced a higher AHI. Schwenger et al. [34] found that patients with NAFLD had a significantly higher AHI and lower minimum oxygen saturation than non-NAFLD patients. Bhatt et al. [35] observed a significant correlation between inflammatory biotests and AHI but didn’t independently test AHI because NAFLD presence was only classified as a binary response (yes/no), similar to OSA—furthermore, Benotti et al. [36] found that AHI and OSA-related outcomes were not significantly correlated in patients with metabolic syndrome, though AHI quartiles increased stepwise in patients with obesity who had lobular inflammation. Aron-Wisnewsky et al. [29] illustrated that in patients with severe obesity, chronic intermittent hypoxia, notably with the highest ODI, was strongly associated with more severe liver injuries. Whereby Corey et al. [37] pointed out that the absence of OSA seems to provide a protective effect against NAFLD in patients with obesity. In the context of differing demographics, Bhatt et al. [35] discovered that systemic inflammation is significantly more pronounced in Asian Indian patients, particularly those with obesity and OSA along with NAFLD. Finally, Kim T. et al. [28] emphasized the close association of OSA with NAFLD in the general population, with a noteworthy observation that the prevalence of NAFLD is more accentuated in patients living with obesity.

## Cohort-studies

Acarturk et al., Polotsky et al., Krolow et al., Byrne et al., Kallwitz et al., and Ulitsky et al. did not test AHI or other OSA-related indicators of NAFLD. These studies only used the AHI for defining OSA [39–41, 43, 45, 47]. Agrawal et al. [42] found that AHI was significantly correlated with NAFLD in multivariate analysis. In addition, ALT and AST levels were correlated with AHI and NAFLD. Weingarten et al. [46] found no relationship between AHI and other OSA-related indicators of NAFLD, which has also been previously described for OSA.

## Results on the relation between OSA and NAFLD

Of all the cross-sectional studies included, 16/18 (89%) found a correlation between obesity in patients with OSA and the development of NAFLD—however, two studies (11%), Turkay et al. [30] from 2012 and Jouët et al. [20] from 2007, found no evidence of a correlation. Further, Jouët et al. found no relationship between OSA and NASH or other liver lesions in patients with severe obesity.

In 8/10 cohort studies (80%) found a correlation between patients with obesity and OSA toward NAFLD—however, two studies, by Acaturk et al. [39] in 2007 and Weingarten et al. [46] in 2012, did not find this.

## Diagnostic tests for accurate NAFLD correlated to OSA

During the process of data extraction and evaluation of liver outcomes, demographic characteristics, and outcome definitions for OSA, the findings revealed substantial heterogeneity, incomplete information, and disparate grading or scoring methodologies for associating NAFLD diagnostic instruments with OSA definitions. We extracted all the diagnostic tests for NAFLD in relation to OSA and identified 14 distinct liver diagnostic tools, in addition to blood sample analysis for liver function (Table 2).

**Table 2:** Overview of variables for diagnosing NAFLD.

Author	NAS score	NAFLD Activity grade	Liver fibrosis score FIB-4	FIN	FLI	Presence of total inflammation	Presence of lobular inflammation	Presence of fibrosis	Steatosis stage	Ballooning stage	Lobular inflammation stage	Fibrosis stages	Fibro inflammation	Liver volume Or Liver/ kidney echo ratio
**Cross-sectional studies**														
Jouët et al.	**-**	**-**	**-**	**-**	**-**	**-**	**-**	**-**	**+**	**-**	**-**	**+**	**-**	**-**
Mishra et al.	**-**	**-**	**-**	**-**	**-**	**-**	**-**	**+**	**-**	**-**	**-**	**-**	**-**	**-**
Campos et al.	**-**	**+**	**-**	**-**	**-**	**-**	**-**	**-**	**-**	**-**	**-**	**-**	**-**	**-**
Daltro et al.	**-**	**-**	**-**	**-**	**-**	**+**	**+**	**+**	**+**	**+**	**-**	**-**	**-**	**-**
Turkay et al.	**-**	**-**	**-**	**-**	**-**	**-**	**-**	**-**	**-**	**-**	**-**	**-**	**-**	**+**
Aron-Wisnewsky et al.	**+**	**-**	**-**	**-**	**-**	**-**	**-**	**-**	**+**	**+**	**+**	**+**	**+**	**-**
Corey et al.	**+**	**-**	**-**	**-**	**-**	**-**	**-**	**-**	**+**	**+**	**+**	**+**	**-**	**-**
Mesarwi et al.	**-**	**+**	**-**	**-**	**-**	**-**	**+**	**-**	**+**	**+**	**-**	**-**	**-**	**-**
Benotti et al.	**-**	**-**	**-**	**-**	**-**	**-**	**+**	**+**	**+**	**-**	**-**	**-**	**-**	**-**
Scartabelli et al.	**-**	**-**	**-**	**-**	**-**	**-**	**-**	**-**	**-**	**-**	**-**	**-**	**-**	**+**
Bhatt et al.	**-**	**-**	**-**	**-**	**-**	**-**	**-**	**-**	**-**	**-**	**-**	**-**	**-**	**+**
Zhang, L et al.	**-**	**-**	**-**	**-**	**-**	**-**	**-**	**-**	**-**	**-**	**-**	**-**	**-**	**+**
Schwenger et al.	**-**	**-**	**-**	**-**	**-**	**+**	**+**	**-**	**-**	**-**	**-**	**-**	**-**	**-**
Tomar et al.	**-**	**-**	**-**	**-**	**-**	**-**	**-**	**-**	**-**	**-**	**-**	**-**	**-**	**-**
Kim, T. et al.	**-**	**-**	**+**	**-**	**-**	**-**	**-**	**-**	**-**	**-**	**-**	**-**	**-**	**-**
Grillo et al.	**-**	**-**	**-**	**-**	**-**	**-**	**-**	**+**	**-**	**-**	**-**	**-**	**-**	**-**
Fu et al.	**+**	**+**	**+**	**+**	-	-	**-**	**-**	**+**	**+**	**+**	**+**	**-**	**-**

Author	NAS score	NAFLD Activity grade	Liver fibrosis score FIB-4	FIN	FLI	Presence of total inflammation	Presence of lobular inflammation	Presence of fibrosis	Steatosis stage	Ballooning stage	Lobular inflammation stage	Fibrosis stages	Fibro inflammation present	Liver volume Or Liver/ kidney echo ratio
Bettini et al.	**-**	**-**	**+**	**-**	**-**	**-**	**-**	**-**	**-**	**-**	**-**	**-**	**-**	**-**
**Cohort studies**														
Kallwitz et al.	**-**	**-**	**-**	**-**	**-**	**-**	**-**	**-**	**+**	**+**	**-**	**+**	**-**	**-**
Acarturk et al.	**-**	**-**	**-**	**-**	**-**	**-**	**-**	**-**	**+**	**-**	**-**	**-**	**-**	**-**
Polotsky et al.	**+**	**+**	**-**	**-**	**-**	**-**	**-**	**-**	**+**	**+**	**+**	**+**	**-**	**-**
Ulitsky et al.	NASH score +	**-**	**-**	**-**	**-**	**-**	**-**	**-**	**-**	**-**	**-**	**-**	**-**	**-**
Byrne et al.	**-**	**-**	**-**	**-**	**-**	**-**	**-**	**-**	**-**	**-**	**-**	**-**	**-**	**-**
Weingarten et al.	**+**	**-**	**-**	**-**	**-**	**-**	**-**	**-**	**+**	**+**	**-**	**+**	**-**	**-**
Agrawal et al.	**-**	**-**	**-**	**-**	**-**	**-**	**-**	**-**	**+**	**-**	**-**	**-**	**+**	**-**
Krolow et al.	**+**	**-**	**-**	**-**	**-**	**-**	**-**	**-**	**+**	**-**	**-**	**+**	**-**	**-**
Zhang, Y.X et al.	**-**	**-**	**-**	**-**	**-**	**-**	**-**	**-**	**-**	**-**	**-**	**-**	**-**	**+**
Chung et al.	**-**	**-**	**-**	**-**	**+**	**-**	**-**	**-**	**-**	**-**	**-**	**-**	**-**	**-**
Total studies *N* = 28 (100%)														
% of NAFLD test used	25%	14%	11%	4%	4%	7%	14%	14%	46%	29%	14%	29%	7%	18%

In summary, the most used tests were steatosis (46%), fibrosis and ballooning (both 29%), and NAS score (25%). The two least used tests were FIN and FLI (both 4%) (Table 2).

### NAS and NAFLD activity

The NAS (NAFLD activity score) was used by 3/18 cross-sectional and 4/10 cohort studies, with different scorings mechanisms from 0-2, 3-4, and >5. Different NAFLD activity grades from 0 to >3 were utilized by 3/18 cross-sectional and 1/10 cohort studies. Cohort studies also used various NASH definitions. Outcome definitions varied across studies, with OSA scored in four categories, not presented as reusable data, or using NAFLD correlation instead of OSA. (Appendix 2).

### Fibrosis 4 index (FIB-4)

The FIB-4 was used in 3/18 CS studies and none of the cohort trials. The outcome definition had two OSA scales, and the FIB-4 index was not presented to reuse the data or correlate with NAFLD instead of OSA.

### Fatty liver index (FLI)

FLI was not used in the CS studies but only in 1/10 cohort studies (Appendix 2).

### Presence of total inflammation

Total inflammation was reported in 2/18 cross-sectional studies and none of the cohort studies. OSA outcome definitions varied between these studies, with age and BMI undefined in the OSA group but generally presented. (Appendix 2).

## Presence of lobular inflammation

Lobular inflammation was reported in 4/18 cross-sectional studies but not in cohort studies. Two had the same OSA outcome definition, but only three reported lobular inflammation. Data correlation was impossible due to inconsistent classification (Appendix 2).

## Presence of fibrosis

Fibrosis was reported in 5/18 cross-sectional studies but not in cohort studies. Two had the same OSA outcome definition, while others had different OSA or NAFLD definitions. One study only classified fibrosis. (Appendix 2).

## Steatosis

Steatosis was a variable in 7/18 cross-sectional and 6/10 cohort studies. Different outcome definitions were used in all seven cross-sectional studies, while two of the six cohort studies shared definitions. Steatosis occurrence wasn’t presented as usable data. (Appendix 2).

## Ballooning

Ballooning was used in 5/18 cross-sectional and 3/10 cohort studies. Two cross-sectional studies shared NAFLD outcome definitions but had different OSA categories. Ballooning correlated with NAFLD, not OSA. Cohort studies had two sharing OSA criteria, but data presentation was limited. (Appendix 2).

## Lobular inflammation

Lobular inflammation was featured in 3/18 CS and 1/10 cohort studies. None shared the same OSA category. One study correlated lobular inflammation with NAFLD, while the cohort study only presented the data in a figure. (Appendix 2).

## Fibrosis

Fibrosis appeared in 4/18 CS and 4 of 10 cohort studies. CS studies had varying outcome definitions, while two cohort studies shared the same OSA category. Additionally, two cohort studies either didn’t present fibrosis or only displayed it in a figure. (Appendix 2).

## Fibroinflammation

Fibroinflammation was used in 1/18 and 1/10 CS and cohort studies, respectively (Appendix 2).

## Liver volume cm^3^ or liver/kidney echo ratio

Liver volume or liver/kidney echo ratio was used in 4/18 cross-sectional and 1/10 cohort studies, with no shared outcome definitions. Two cross-sectional studies did not present actual results, only general categorical expressions of NAFLD echo. (Appendix 2).

### Effects of CPAP on OSA and NAFLD

In total, four CS studies and one cohort study described CPAP use. Mesarwi et al. [23] found that hypoxic stress in OSA increases circulating lysyl oxidase (LOX) levels. However, LOX was decreased in patients who used CPAP compared to those who did not. Thus, LOX may serve as a biomarker of liver fibrosis in patients with severe obesity and NAFLD, and CPAP may be an effective approach.

The study by Mishra et al. [24] found that, in patients with NALFD, a higher mean CPAP pressure requirement was necessary compared to the non-NAFLD group (10.6 vs. 8.6 mmHg, *P* = 0.04), which may indicate that patients with worse NAFLD had more nocturnal hypoxic episodes. Nevertheless, no significant difference was found in the requirement for CPAP therapy between patients with NAFLD and non-NAFLD (11% vs. 9%, *P* = 0.18).

Campos et al. [25] described the use of CPAP in the methods section but did not further mention or analyze it in the study. The study by Corey et al. [37] was unable to validate CPAP use or compliance and so it was not measured or corrected for in the statistical analysis. The cohort study by Weingarten et al. [46] found that among BMS patients preoperatively assessed to have OSA and prescribed CPAP when indicated (79.1% were compliant) the severity of OSA was not associated with the severity of NAFLD.

## Discussion

Compared to the previous SRs published in 2013 [3, 4], 14 new publications were published and included in our SR together with the previous studies before 2013. Additionally, it is the first SR to investigate the effects and evaluation of OSA-related outcomes, as well as the assessment of associations in diagnostic tests employed for accurately measuring NAFLD in patients with obesity. This study elucidates the relationship between OSA, NAFLD, and BMS. Only one study assessed the impact of OSA on NAFLD, considering the influence of substantial follow-up periods before and after BMS, and observing significant liver outcomes over time. The majority of the studies were cross-sectional, lacking follow-up assessments.

### Effects of OSA-related outcomes and NAFLD

In 66% of the cross-sectional studies examined, a significant correlation between OSA-related outcomes and NAFLD was observed, while 14% found no difference, and 20% did not assess this outcome. In cohort studies, 75% did not evaluate the relationship between OSA-related outcomes and NAFLD, 13% identified significant differences, and 13% found no differences. Consequently, several studies did not measure OSA-related outcomes in relation to NAFLD, potentially underestimating their effects and reducing the reliability of conclusions.

Oxidative stress induced by hypoxemia is implicated as a critical factor in NAFLD progression, as posited by the “second hit” hypothesis. It was noted that many studies did not measure or test AHI indicators for OSA. AHI alone was found to be an insufficient predictor for NAFLD; however, the cascade of effects resulting from OSA significantly contributed to an increased prevalence of NAFLD. Thus, OSA-related outcomes, such as desaturation, appear to be the primary connection to alterations in liver status among patients with obesity.

Given the inadequacy of AHI as a predictor for NAFLD, future studies should emphasize OSA-related outcomes more. As weight loss occurs and recovery from associated medical problems like OSA progresses, AHI decreases; however, the impact of hypoxemia should still be evaluated within the remaining AHI. In the study by Zhang et al. [44], neither baseline characteristics, OSA, nor the extent of weight loss was the most critical factor for NAFLD improvement. Instead, respiratory and metabolic changes led to reduced oxidative stress induced by hypoxemia. Since almost all the included studies lacked follow-up time, it is difficult to assess the effect of time on the disease state of OSA for increased NAFLD. It is unclear after what period of OSA the stages of NAFLD start. Since higher stages are irreversible, this is important to know. Furthermore, non-contrast CT, a reliable non-invasive imaging method, to detect moderate or severe hepatic steatosis at baseline and 6 months after surgery was used by Zhang et al. [44]. While this does not provide the same level of detail as a biopsy, it does allow for the detection of changes in liver fat content. While the results suggest that BMS may benefit NAFLD, further research, ideally with histological and functional data, is needed to confirm these findings. Therefore, well-controlled cohort studies with longitudinal measurements must elucidate these pathways and identify which conditions require treatment first to mitigate NAFLD or which conditions demand monitoring postoperatively.

A low AHI accompanied by persistent desaturations may continue to impact NAFLD negatively. In this systematic review, only 1/28 studies investigated the influence of NAFLD and OSA in a follow-up study. Neglecting to assess these factors or failing to examine their impact over time may not provide adequate information regarding long-term effects, potentially compromising the accuracy of scientific conclusions.

Ultimately, individual studies focusing on OSA versus BMS and NAFLD versus BMS produce distinct results. Future research should further explore the effect of BMS on these subclassified variables to reveal new associations with NAFLD specific to OSA-related outcomes, which could serve as potential predictors.

### Impact of BMS

Before 2020 no studies are known on the effect of BMS in relation to OSA and NAFLD in the same cohort. Nevertheless, multiple SRs showed that only OSA generally decreases or fully recovers after BMS [48–50]. Other SRs showed that weight loss after BMS in only NAFLD generally improves NAFLD [5–11].

A comprehensive evaluation of the relationship between Bariatric and Metabolic Surgery (BMS) and Non-Alcoholic Fatty Liver Disease (NAFLD) in 2010 revealed that the systematic review (SR) failed to identify randomized clinical trials or quasi-randomized clinical studies. Instead, it discovered twenty-one prospective or retrospective cohort studies reporting improvements in steatosis or inflammation scores. Notably, four studies also documented some deterioration in fibrosis levels. The non-randomized controlled trial (non-RCT) designs of these studies limited the ability to draw unbiased conclusions regarding BMS as a treatment for NAFLD. In 2010, Roux-en-Y gastric bypass (RYGB) was the most common BMS for treating NAFLD. However, by 2021, the most frequently conducted surgery was laparoscopic sleeve gastrectomy (LSG), followed by RYGB, with the biliopancreatic diversion with duodenal switch and gastric banding being relatively rare procedures. The findings suggest that BMS may be a viable option for carefully selected patients with obesity and cirrhosis, although these patients may experience slightly elevated morbidity and mortality rates. RYGB yielded the most significant improvements in steatosis, while LSG led to the most notable ameliorations in fibrosis. These results strongly imply that BMS should be considered as a treatment for NAFLD. BMS appeared safe and associated with acceptable perioperative and long-term outcomes for patients with severe obesity and cirrhosis with portal hypertension.

However, all SRs emphasized the need for higher levels of evidence to determine the benefits of BMS on liver disease and to refine future treatment strategies for managing NAFLD. More robust, evidence-based recommendations are necessary, as there is currently a lack of consensus on managing patients with obesity and the safety and efficacy of BMS in this population. The design and follow-up limitations in existing studies obscure the safety and benefits of BMS for patients with cirrhosis. This SR discussed that even when the effects of BMS on Obstructive Sleep Apnea (OSA) and NAFLD alone were tested, we can only assume that both effects impact the improvement of OSA and NAFLD. This SR found that only one study investigated the effects of OSA and NAFLD before and after BMS with an extensive follow-up period, confirming these assumptions.

### OSA and NAFLD in patients with and without obesity

In our systematic review, we primarily investigated the impact of OSA on NAFLD in patients with obesity. When we expanded our analysis to include studies involving patients with no or overweight the correlation became more defined.

For instance, Ding et al. established a link between OSA and NAFLD. Notably, they identified intermittent hypoxia, a consequence of OSA, as an independent predictor for NAFLD among severely OSA-afflicted patients. This held true across a BMI range of 24.09–27.92, encompassing overweight individuals [51]. On the other hand, Qi et al.‘s study presents an intriguing contrast. Their findings suggest that in individuals with an average or slightly above-average BMI (range 23.71–24.14), OSA does not significantly affect liver enzymes [52]. However, they highlighted the importance of lipid metabolism, weight management, and OSA-induced hypoxemia in mitigating the risk of NAFLD among OSA patients.

This brings to light a crucial perspective: the definition of OSA, based on the Apnea-Hypopnea Index (AHI), may not be the only determinant. OSA-related outcomes like hypoxemia, induced by apnea or hypopnea, could also be significant contributors.

Further supporting this notion, two studies by Tatsumi et al. [53]. and Tanné et al. [54]. postulate that recurrent hypoxemia in OSA patients, particularly those with hepatic steatosis, might pose a risk for the development of NASH and could trigger insulin resistance. This underscores the weight-independent effects of OSA on liver health.

Our systematic review indicated a relationship between OSA and NAFLD in patients with obesity. However, we recognize that our exploration of this relationship in patients who have no obesity or are only slightly overweight was not as methodologically rigorous. Preliminary findings suggest OSA’s impact on the liver may be independent of weight, but further investigations are required. Our results highlight the importance of considering OSA when performing BMS in patients with obesity and focus on the adverse effects of OSA (hypoxia) instead of the OSA definition itself. Future research should investigate OSA’s impact on NAFLD across different BMI categories to inform tailored treatment strategies.

### CPAP and NAFLD

The data on the effectiveness of CPAP in managing NAFLD is still inconclusive, as indicated by the four case series and one cohort study in our review. Of these, only one study demonstrated a significant effect of CPAP, which aligns with findings from a previous systematic review by Chen et al. [55] shows decreased liver enzymes in patients with OSA using CPAP.

However, both our review and the previous work by Chen et al. highlight the lack of comprehensive assessment of NAFLD, especially using specific measures such as the NAFLD activity score. It’s important to remember that AST and ALT, while widely used, may not provide a complete picture of NAFLD progression or remission.

These findings underscore the complexity of the relationship between CPAP use and NAFLD outcomes, which might be influenced by factors such as patient adherence to therapy, severity of OSA, and co-existing metabolic conditions.

The real effect of CPAP on NAFLD remains to be fully elucidated, and our review highlights a crucial gap in the current understanding. Future research should employ more accurate and specific diagnostic tests for NAFLD to provide a more comprehensive insight into the potential therapeutic effects of CPAP on this condition.

### Diagnostic tests for NAFLD

If we look at the designs of the two previous SRs from Musso et al. and Sookoian et al. from 2013, both performed meta-analyses of the extracted data [3, 4]. Using appropriate methodological techniques and achieving low heterogeneity after the meta-analysis provides a comprehensive understanding of the outcomes of OSA and NAFLD.

Nevertheless, after we reviewed the data and exposed all liver value techniques divided between the two study designs, we have some concerns about whether the pooling of the data was justified in the used outcome values and study design.

Because this SR highlights the shortcomings of the possible diagnostic ability of NAFLD. Out of the 14 different tests for NAFLD that were described as the “gold standard”, “NAS”, or “NAFLD activity score”, only 3 out of 18 cross-sectional studies and 4 out of 10 cohort studies used the NAS. Only 3 out of 18 cross-sectional and 1 out of 10 cohort studies used the NAFLD activity score. In addition, several different cut-off values were used within each test, and data were not presented as a score or grade. To provide a comprehensive overview of the diagnosis or treatment of NAFLD, it is essential to ensure uniformity in the outcomes and to make the data readily available for review or use in meta-analyses.

For these reasons, conducting a meta-analysis in this SR was not justified, as different study designs should always be analyzed separately, which was not done in the previous SR.

The question remains whether determining a NAFLD, at whatever stage it is, is sufficiently accurate when 14 different diagnostic tests are used and, within these tests, different cutoff values. It would be recommended for uniformity and allowability that there be a standard set of data variables to determine NAFLD within the field of BMS. This SR also noted that the way of presenting data was not done in a univocal or easily usable way.

As scientists, presenting data with high accuracy is beneficial, particularly when we aim to make conclusions about mixed study results. Given the limited sensitivity of current biomarkers for accurate diagnosis of NAFLD and fibrosis, liver biopsy remains necessary to precisely assess histological features. Even when all individual tests are validated, they should be re-evaluated for proper research and diagnosis, with a consensus on which markers to use in the field of BMS. This is particularly relevant given the low level of evidence following BMS, as correlations between OSA-related outcome markers are lacking. Consequently, it is challenging to determine which NAFLD marker best predicts patient outcomes in relation to BMS and hypoxia in OSA.

Multiple variables generate confounding effects, making it difficult to control for potential biases and leaving hypotheses unresolved. Therefore, future research should identify and validate reliable biomarkers for NAFLD and fibrosis while considering the complex interplay between variables such as BMS, OSA, and hypoxia. Developing a consensus on the appropriate markers to use and understanding their interactions will be crucial for enhancing diagnostic accuracy and informing personalized treatment strategies for patients with NAFLD and OSA.

### Risk of bias

The risk of bias (RoB) assessment revealed that cross-sectional studies exhibited a low RoB, indicating a higher quality of the studies and their results. However, the cross-sectional design, capturing only a snapshot of the situation, may lack sufficient scientific power to determine long-term effects. This design is well-suited for identifying NAFLD cases if the samples chosen accurately represent the population and if the appropriate test is employed. Nevertheless, the optimal choice of test remains debated, as demonstrated by the high heterogeneity and inconsistency across studies.

The cohort study design exhibited a higher RoB, particularly during follow-up visits (80% high RoB). This issue could be addressed by adopting novel study designs. The fact that 9 out of 10 studies did not assess OSA and NAFLD before and after surgery highlights an area requiring improvement. To enhance the quality and reliability of future research, it is crucial to employ more robust study designs, conduct comprehensive pre- and post-surgery evaluations of OSA and NAFLD, and establish consensus on appropriate diagnostic tests. This will ultimately lead to a better understanding of the complex relationships between OSA, NAFLD, and bariatric surgery outcomes, and inform more effective treatment strategies for patients.

## Conclusion

In most studies, specific OSA-related outcomes, such as Oxygen Desaturation Index Lowest Arterial Oxygen Saturation, Mean Oxygen Saturation, Nadir Oxygen Saturation, and the percentage of Sleep Time with Oxygen Saturation below 90% measurements for oxidative stress induced by hypoxemia, were significantly correlated with NAFLD. In contrast, the general Apnea-Hypopnea Index (AHI) had a less predictive capacity.

For the first time, BMS has been tested in treating both OSA and NAFLD pre and postoperative with positive results. Further research, ideally with histological and functional data, is needed to confirm these findings. The SR identified 14 different liver outcome tests, but high heterogeneity and incomplete data precluded a meta-analysis. It is crucial to devote greater attention to the impact of OSA-related factors and uniformity in liver outcomes testing for NAFLD. To achieve this, study designs should be enhanced by incorporating more comprehensive pre- and postoperative evaluations, extending follow-up periods, and adopting a more consistent methodology for liver diagnosis in patients with obesity.

### Supplementary information

Appendix 1

Appendix 2
