# Supplementary material for: Effects of obstructive sleep apnea on non-alcoholic fatty liver disease in patients with obesity: a systematic review
Source: Int J Obes (Lond). 2023 Sep 11;47(12):1200–13. doi: 10.1038/s41366-023-01378-2 (PMC10663145; doi:10.1038/s41366-023-01378-2)
Supplement: Supplementary file 2 — Appendix 2 [file 41366_2023_1378_MOESM2_ESM.pdf]

Appendix 2: Stratification of Liver outcomes and demographics characteristics

----

2.1 Cross-sectional studies NAS score

**Author**<br>**n= (%)**	**Outcome definition**	**Gender**<br>**Female**	**Age**	**BMI**	**AST|ALT**	**NAS score 0-2**	**NAS score 3-4**	**NAS >5**
Aron-Wisnewsky et al.	Mild OSA<br>Moderate OSA<br>Severe OSA	Not presented	43.5±9.7<br>41.4±11.5<br>48.4±11.3	45.7±5.7<br>46.6±6.8<br>48.3±7.0	21.7±7.8|30.4±19.3<br>22.9±8.4|29.7±20.9<br>23.3±6.6|33.6±16.3	25 (75.8%)<br>21 (61.8%)<br>16 (47.1%)	7 (21.2%)<br>9 (26.5%)<br>4 (11.8%)	1 (3%)<br>4 (11.8%)<br>3 (8.8%)
Fu et al.	No OSA<br>Mild OSA<br>Moderate/ Severe OSA	§ 94 (70.1%)<br>§§ 23 (46.9%)	§30.9±0.8<br>§§31.7±1.4	§41.1±0.5<br>§§ 41.8±0.9	31.7±3.1|49.2±4.3<br>38.4±3.8|64.3±7.0<br>46.9±4.1|84.0±9.0	27 (30%)<br>9 (19.1%)<br>3 (6.5%)	48 (53.5%)<br>25 (54.2%)<br>22 (47.8%	15 (16.7%)<br>13 (27.7%)<br>21 (45.7%)
Corey et al.	Non-NAFLD<br>NAFLD	45 (91.8%)<br>87 (79.1%)	40.3±10.4<br>47.8±10.4	45.7±5.5<br>47.5±8.6	- | 16.9±7.9<br>- | 28.3±27.1	-<br>49 (44.5%)	-<br>33 (30.0%)	-<br>28 (25.5%)

§ gender, age and BMI expressed to non-NAFLD not to OSA
§§ gender, age and BMI expressed to NAFLD not to OSA

2.1 Cohort studies NAS score

Author<br>n= (%)	Outcome definition	Gender<br>Female	Age	BMI	AST|ALT	NAS score<br>0-2	NAS score<br>3-4	NAS >5
Polotsky et al.	Not presented<br>But only used in figures	# 75 (83.3%)	# 41.1±9.5	#49.0±7.9	*17.5±6.6|11.1±6.1<br>**17.8±5.9|12.3±6.2	-	-	-
						**NASH score No**	**NASH score Mild**	**NASH score Advanced**
Weingarten et al.	No/mild OSA<br><br>Moderate/severe OSA	106 (84.8%)<br><br>63 (67.7%)	47.7±11.1<br><br>48.9±10.4	47.0±7.6<br><br>49.2±10.8	28.0±11.0|36.0±24<br><br>28.0±13.0|38.0±19.0	55 (44.0%)<br><br>39 (41.9%)	50 (40.0%)<br><br>44 (47.3%)	20 (16.0%)<br><br>10 (10.8%)
						**NASH score No**	**NASH Score Yes**	-
Ulitsky et al.	OSA no<br>OSA yes	§175 (87.0%)<br>§§44 (84.6%)	§ 42.3± -<br>§§46.6±-	§ 48.1± -<br>§§ 48.7± -	§19.6 | 24.3<br>§§ 19.9 | 26.8	-<br>64 (31.8%)	 28 (53.8%)	-
						-	**NASH mild**	**NASH moderate**
Krolow et al.	No/mild OSA<br>Moderate/severe OSA	21 (51.2%)<br>5 (50.0%)	57.0±8.2<br>58.7±7.0	# 33.0±5.3	& 4 (9.8%) | 17 (41.5%)<br>& 2 (20.0%) | 3 (30.0%)	-	36 (75.0%)<br>12 (25.0%)	29 (76.3%)<br>9 (23.7%)

In general, not specific to a group
*Correlated to mild OSA ** moderate OSA
§ gender, age and BMI expressed to non-NAFLD not to OSA
§§ gender, age and BMI expressed to NAFLD not to OSA
& Alternated AST and ALT expressed in n (%), no references values

----

2.2 Cross-sectional studies NAFLD activity

**Author**<br>**n= (%)**	**Outcome definition**	**Gender Female**	**Age**	**BMI**	**AST|ALT**	**NAFLD activity Grade 0**	**NAFLD activity Grade 1**	**NAFLD activity Grade 2**	**NAFLD activity Grade >3**
Mesarwi et al.	Non-NAFLD<br>NAFLD	15 (79.0%)<br>11 (69.0%)	45.2±10.4<br>53.1±7.5	46.7±6.7<br>50.2±5.4	23.8±13.0 |29.9±43.4<br>23.8±12.0 |26.0±28.0	*1.6±0.3<br>*3.4±2.2	-	-	-
Campos et al.	Non-NAFLD<br>NAFLD	118 (86.8%)<br>50 (78.1%)	42 (36-50)<br>44 (38-54)	48 (43-54)<br>50 (44-57)	20(18-25) | 22(18-30)<br>28(23-35) | 34(26-42)	-<br>Not presented	-<br>Not presented	-<br>Not presented	-<br>Not presented
Fu et al.	No OSA<br>Mild OSA<br>Moderate/ Severe OSA	§ 94 (70.1%)<br>§§ 23 (46.9%)	§30.9±0.8<br>§§31.7±1.4	§41.1±0.5<br>§§ 41.8±0.9	31.7±3.1 | 49.2±4.3<br>38.4±3.8 | 64.3±7.0<br>46.9±4.1 | 84.0±9.0	3 (3.3%)<br>0 (0.0%)<br>1 (2.2%)	44 (48.9%)<br>20 (42.6%)<br>7 (15.2%)	31(34.4%)<br>21 (44.7%)<br>17 (37.0%)	12 (13.3%)<br>6 (12.7%)<br>21 (45.6%)

§ gender, age and BMI expressed to non-NAFLD not to OSA
§§ gender, age and BMI expressed to NAFLD not to OSA
*Total NAFLD activity score (not classified in grade scores)

2.2 Cohort studies NAFLD activity

**Author**<br>**n= (%)**	**Outcome definition**	**Gender Female**	**Age**	**BMI**	**AST|ALT**	**NAFLD activity Grade 0**	**NAFLD activity Grade 1**	**NAFLD activity Grade 2**	**NAFLD activity Grade >3**
Polotsky et al.	Mild OSA<br>Moderate/severe OSA	42 (93.3%)<br>33 (73.3%)	38.8±8.1<br>43.3±10.2	48.1±7.9<br>49.9±7.9	17.5±6.6 | 11.1±6.1<br>17.8±5.9 | 12.3±6.2	Not presented Only classified in figure	-	-	-

In general, not specific to a group
*Correlated to mild OSA, and ** moderate OSA

2.3 Cross-sectional studies FIB-4

**Author Mean±sd**	**Outcome definition**	**Gender Female**	**Age**	**BMI**	**AST|ALT**	**Liver fibrosis score (FIB-4)**
Bettini et al.	No OSA<br>Mild OSA<br>Moderate OSA<br>Severe OSA	45 (85.6%)<br>60 (68.2%)<br>32 (49.2%)<br>39 (31.5%)	41.1±14.1<br>48.8±10.6<br>50.2±10.8<br>49.8±12.2	40.7 (36-47.5)<br>41 (37.7-47.9)<br>44.3 (40.1-49.2)<br>45.2 (39.6-51.2)	23 (18-32) |23 (16-31)<br>23 (19-32) | 23 (18-37)<br>26 (21-32) | 26 (20-32)<br>29 (24-42) | 30 (21-40)	0.79 (0.48-1.26)<br>0.97 (0.73- 1.42)<br>1.07 (0.74-1.48)<br>1.20 (0.91-1.85)
Kim, T. et al.	Non-NAFLD<br>NAFLD	1646 (50.6%)<br>442 (43.3%)	55.6±0.3<br>53.9±0.4	* 722 (22.2%)<br>*878 (86.3%)	23.4±0.2|19.1±0.2<br>27.3±0.4|36.5±0.8	Not presented
Fu et al.	No OSA<br>Mild OSA<br>Moderate/ Severe OSA	§ 94 (70.1%)<br>§§ 23 (46.9%)	§30.9±0.8<br>§§31.7±1.4	§41.1±0.5<br>§§ 41.8±0.9	31.7±3.1|49.2±4.3<br>38.4±3.8|64.3±7.0<br>46.9±4.1|84.0±9.0	# 0.6±0.0<br>## 0.6±0.0

Correlated to non-NAFLD, not too OSA
Correlated to NAFLD, not too OSA
*BMI in categories >25 kg/m2 presented

Not used in cohort studies FIB-4

Not used in Cross-sectional studies Fatty liver index (FLI)

2.4 Cohort studies Fatty liver index (FLI)

**Author Mean±sd**	**Outcome definition**	**Gender Female**	**Age**	**BMI**	**AST|ALT**	**Fatty liver index (FLI)**
Chung et al.	Fatty liver index 0-30	3.165.384 (59.1%)	§ 20-39 (35.4%)<br>40-64 (52.8%)<br>≥65 (11.8%)	22.21 ± 2.41	Not present	5,352,484 (50.9%)
	Fatty liver index 30-60	562.749 (30.7%)	§ 20-39 (24.3%)<br>40-64 (59.0%)<br>≥65 (16.7%)	25.52 ± 2.24	Not present	1,833,489 (17.5%)
	Fatty liver index >60	173.641 (18.7%)	§ 20-39 (30.3%)<br>40-64 (58.2%)<br>≥65 (11.5%)	27.93 ± 2.99	Not present	930,551 (8.6%)

§Age categorized

2.5 Cross-sectional studies Presence of total inflammation

**Author**<br>**n= (%)**	**Outcome definition**	**Gender**<br>**Female**	**Age**	**BMI**	**AST|ALT**	**Presence of total inflammation**
Daltro et al.	Mild OSA<br>Moderate/severe OSA	15 (93.6%)<br>5 (31.2%)	#36.2±9.6	#41.6±4.7	* 0.0% | 21.7%<br>* 12.5% | 37.5%	19 (79.2%)<br>13 (81.2%)
Schwenger et al.	No OSA<br>Mild OSA<br>Moderate OSA<br>Severe OSA	§ 10 (83.0%)<br>§§ 33 (67.0%)	§ 47.8 (40-55)<br>§§ 48.1 (43-56)	§ 49.42 (42-55)<br>§§ 49.82 (43-55)	§ 18.1(16.5-20) | 22.1(15-26)<br>§§ 42.6 (17-36) | 35.0 (21-47)	5 (45.5%)<br>5 (62.5%)<br>9 (90.0%)<br>17 (85.0%)

In general, not specific to a group

*Elevated AST and AHI in percentage (no description of when elevated occurred)

§ Correlated to non-NAFLD, not too OSA classes in mean (first quartile, third quartile)

§§ Correlated to NAFLD, not too OSA classes in mean (first quartile, third quartile)

Not used in cohort studies Presence of total inflammation

2.6 Cross-sectional studies Presence of lobular inflammation

**Author n= (%)**	**Outcome definition**	**Gender Female**	**Age**	**BMI**	**AST|ALT**	**Presence of lobular inflammation**
Daltro et al.	Mild OSA<br>Moderate/severe OSA	15 (62.5%)<br>5 (31.2%)	#36.2±9.6	#41.6±4.7	* 0.0% | 21.7%<br>* 12.5% | 37.5%	19 (79.2%)<br>13 (81.2%)
Mesarwi et al.	Non-NAFLD<br>NAFLD	15 (79.0%)<br>11 (69.0%)	45.2±10.4<br>53.1±7.5	46.7±6.7<br>50.2±5.4	23.8±13.0 |29.9±43.4<br>23.8±12.0 |26.0±28.0	**0.6±0.5<br>**1.1±0.7
Schwenger et al.	No OSA<br>Mild OSA<br>Moderate OSA<br>Severe OSA	§ 10 (83.0%)<br>§§ 33 (67.0%)	§ 47.8 (40-55)<br>§§ 48.1 (43-56)	§ 49.42 (42-55)<br>§§ 49.82 (43-55)	§ 18.1(16.5-20) | 22.1(15-26)<br>§§ 42.6 (17-36) | 35.0 (21-47)	3 (27.3%)<br>3 (37.5%)<br>8 (80.0%)<br>15 (75.0%)
Benotti et al.	No OSA<br>Mild OSA<br>Moderate OSA<br>Severe OSA	83 (89.0%)<br>100 (87.0%)<br>64 (80.0%)<br>40 (54.0%)	41.3±9.5<br>46.7±11.0<br>48.8±10.7<br>48.7±10.5	46.8±7.5<br>48.9±8.9<br>50.9±9.0<br>54.2±11.0	26.2±11.3 | 30.0±11<br>25.3±11.5 | 28.8±11.5<br>28.5±15.6 | 32.5±15.6<br>28.6±23.4 | 35.2±23.4	Not presented<br>Only classified in figure

In general, not specific to a group
*Elevated AST and AHI in percentage (no description of when elevated occurred)
**Lobular inflammation score (not classified in presence scores)
§ Correlated to non-NAFLD, not too OSA classes in mean (first quartile, third quartile)
§§ Correlated to NAFLD, not too OSA classes in mean (first quartile, third quartile)
Not used in cohort studies

2.7 Cross-sectional studies Presence of fibrosis

**Author n= (%)**	**Outcome definition**	**Gender Female**	**Age**	**BMI**	**AST|ALT**	**Presence of fibrosis**
Daltro et al.	Mild OSA<br>Moderate/severe OSA	15 (93.6%)<br>5 (31.2%)	#36.2±9.6	#41.6±4.7	* 0.0% | 21.7%<br>* 12.5% | 37.5%	16 (66.7%)<br>13 (81.2%)
Mishra et al.	Non-NAFLD<br>NAFLD	17 (77.3%)<br>55 (69.6%)	37.9±12.9<br>44.2±10.6	47.3±7.7<br>52.6±9.6	21.8±9.2 | 26.4±11.3<br>25.3±16.6 | 34.4±25.0	0 (0.0%)<br>73 (92.4%)
Grillo et al.	Mild OSA<br>Severe OSA	25 (64.1%)<br>74 (63.8%)	55.7±8.9<br>55.2±11.0	29.0±3.4<br>31.6±5.0	32.6±22.7 |42.7±35.3<br>33.5±19.2 | 42.7±28.7	15 (38.5%)<br>73 (62.9%)
Schwenger et al.	No OSA<br>Mild OSA<br>Moderate OSA<br>Severe OSA	§ 10 (83.0%)<br>§§ 33 (67.0%)	§ 47.8 (40-55)<br>§§ 48.1 (43-56)	§ 49.42 (42-55)<br>§§ 49.82 (43-55)	§ 18.1(16.5-20) | 22.1(15-26)<br>§§ 42.6 (17-36) | 35.0 (21-47)	6 (54.5%)<br>7 (87.5%)<br>6 (60.0%)<br>16 (80.0%)
Benotti et al.	No OSA<br>Mild OSA<br>Moderate OSA<br>Severe OSA	83 (89.0%)<br>100 (87.0%)<br>64 (80.0%)<br>40 (54.0%)	41.3±9.5<br>46.7±11.0<br>48.8±10.7<br>48.7±10.5	46.8±7.5<br>48.9±8.9<br>50.9±9.0<br>54.2±11.0	26.2±11.3 | 30.0±11<br>25.3±11.5 | 28.8±11.5<br>28.5±15.6 | 32.5±15.6<br>28.6±23.4 | 35.2±23.4	Not presented<br>Only classified in figure

In general, not specific to a group

*Elevated AST and AHI in percentage (no description of when elevated occurred)

§ Correlated to non-NAFLD, not too OSA classes in mean (first quartile, third quartile)

§§ Correlated to NAFLD, not too OSA classes in mean (first quartile, third quartile)

Not used in cohort studies Presence of fibrosis

2.8 Cross-sectional studies Steatosis

**Author**<br>**n= (%)**	**Outcome definition**	**Gender Female**	**Age**	**BMI**	**AST|ALT**	**Steatosis stage 0**	**Steatosis stage 1**	**Steatosis stage 2**	**Steatosis stage 3**
Jouët et al.	No OSA<br>Moderate OSA<br>Severe OSA	8 (88.9%)<br>32 (86.5%)<br>12 (92.3%)	30.9±11.5<br>38.2±11.3<br>44.5±5.9	45.5±5.5<br>46.7±7.4<br>54.6±9.8	** 23±15 | 45±18	**<br>0 (0.0%)	** 34 (54.8%)	** 14 (22.6%)	** 3 (4.8%)
Daltro et al.	Mild OSA<br>Moderate/severe OSA	15 (93.6%)<br>5 (31.2%)	#36.2±9.6	#41.6±4.7	* 0.0% | 21.7%<br>* 12.5% | 37.5%	0 (0.0%)<br>0 (0.0%)	19 (79.2%)<br>14 (87.5%)	0 (0.0%)<br>0 (0.0%)	0 (0.0%)<br>0 (0.0%)
Mesarwi et al.	Non-NAFLD<br>NAFLD	15 (79.0%)<br>11 (69.0%)	45.2±10.4<br>53.1±7.5	46.7±6.7<br>50.2±5.4	23.8±13.0 |29.9±43.4<br>23.8±12.0 |26.0±28.0	***0.9±0.7<br>***1.6±1.0	-	-	-
Aron-Wisnewsky et al.	Mild OSA<br>Moderate OSA<br>Severe OSA	Not presented	43.5±9.7<br>41.4±11.5<br>48.4±11.3	45.7±5.7<br>46.6±6.8<br>48.3±7.0	21.7±7.8|30.4±19.3<br>22.9±8.4|29.7±20.9<br>23.3±6.6|33.6±16.3	12 (36.4%)<br>6 (17.7%)<br>5 (14.7%)	9 (27.3%)<br>11 (32.4%)<br>7 (20.6%)	7 (21.2%)<br>7 (20.6%)<br>14 (41.2%)	5 (15.2%)<br>10 (29.4%)<br>8 (23.5%)
Fu et al.	No OSA<br>Mild OSA<br>Moderate/ Severe OSA	§ 94 (70.1%)<br>§§ 23 (46.9%)	§30.9±0.8<br>§§31.7±1.4	§41.1±0.5<br>§§ 41.8±0.9	31.7±3.1|49.2±4.3<br>38.4±3.8|64.3±7.0<br>46.9±4.1|84.0±9.0	-<br>-<br>-	45 (50.0%)<br>15 (31.9%)<br>16 (34.85)	22 (24.4%)<br>14 (29.8%)<br>14 (30.4%)	16 (34.8%)<br>14 (30.4%)<br>16 (34.8%)
Benotti et al.	No OSA<br>Mild OSA<br>Moderate OSA<br>Severe OSA	83 (89.0%)<br>100 (87.0%)<br>64 (80.0%)<br>40 (54.0%)	41.3±9.5<br>46.7±11.0<br>48.8±10.7<br>48.7±10.5	46.8±7.5<br>48.9±8.9<br>50.9±9.0<br>54.2±11.0	26.2±11.3 | 30.0±11<br>25.3±11.5 | 28.8±11.5<br>28.5±15.6 | 32.5±15.6<br>28.6±23.4 | 35.2±23.4	Not presented<br>Only classified in figure	-	-	-
Corey et al.	Non-NAFLD<br>NAFLD	45 (91.8%)<br>87 (79.1%)	40.3±10.4<br>47.8±10.4	45.7±5.5<br>47.5±8.6	- | 16.9±7.9<br>- | 28.3±27.1	-<br>12 (10.9%)	-<br>61 (55.5%)	-<br>25 (22.7%)	-<br>12 (10.9%)

*Elevated AST and AHI in percentage (no description of when elevated occurred)
** In general, not specific to a group
*** Steatosis score (not classified in stages)

2.8 Cohort studies Steatosis

Author<br>n= (%)	Outcome definition	Gender Female	Age	BMI	AST|ALT	Steatosis stage 0	Steatosis stage 1	Steatosis stage 2	Steatosis stage 3
Acarturk et al.	No OSA<br>Present OSA	25 (100%)<br>20 (100%)	44.3±10.1<br>50.0±8.5	39.0±6.2<br>39.9±8.0	20.1±8.5 | 22.8±11.0<br>19.7±3.9 | 21.1±5.3	§22 (88.0%)<br>§15 (75.05)	-	-	-
Kallwitz et al.	Mild OSA<br>Moderate/severe OSA	37 (87.0%)<br>26 (60.0%)	42.8±12.1<br>44.5±9.7	51.7±8.5<br>57.2±12.6	*26±15| 35±27	* 1(1.0%)	*29 (34.0%)	*29 (34.0%)	26 (31.0%)
Polotsky et al.	Mild OSA<br>Moderate/severe OSA	42 (93.3%)<br>33 (73.3%)	38.8±8.1<br>43.3±10.2	48.1±7.9<br>49.9±7.9	17.5±6.6 | 11.1±6.1<br>17.8±5.9 | 12.3±6.2	Not presented Only classified in figure	-	-	-
Krolow et al.	No/mild OSA<br>Moderate/severe OSA	21 (51.2%)<br>5 (50.0%)	57.0±8.2<br>58.7±7.0	* 33.0±5.3	& 4 (9.8%) | 17 (41.5%)<br>& 2 (20.0%) | 3 (30.0%)	0 (0.0%)<br>0 (0.0%)	10 (26.3%)<br>6 (60.0%)	14 (36.8%)<br>3 (30.0%)	14 (36.8%)<br>1 (10.05)
Agrawal et al.	NAFLD<br>OSA present	38 (38.0%)<br>5 (22.0%)	41.0±11.0<br>46.0±12.0	28.3±4.4<br>32.2±7.6	45 (33-66) | 71 (44-96)<br>26 (24-44) | 41 (27-64)	-<br>2 (9.0%)	57 (57.0%)<br>8 (35.0%)	32 (32.0%)<br>9 (39.0%)	11 (11.0%)<br>4 (17.0%)
Weingarten et al.	No/mild OSA<br>Moderate/severe OSA	106 (84.8%)<br>63 (67.7%)	47.7±11.1<br>48.9±10.4	47.0±7.6<br>49.2±10.8	28.0±11.0|36.0±24<br>28.0±13.0|38.0±19.0	Not presented	-	-	-

(* In general, not specific to a group) (§ only percentage present, not specified)

2.9 Cross-sectional studies Ballooning

**Author n= (%)**	**Outcome definition**	**Gender Female**	**Age**	**BMI**	**AST|ALT**	**Ballooning stage 0**	**Ballooning stage 1**	**Ballooning stage 2**
Daltro et al.	Mild OSA<br>Moderate/severe OSA	15 (93.6%)<br>5 (31.2%)	#36.2±9.6	#41.6±4.7	* 0.0% | 21.7%<br>* 12.5% | 37.5%	0 (0.0%)<br>0 (0.0%)	22 (91.7%)<br>15 (93.8%)	0 (0.0%)<br>0 (0.0%)
Mesarwi et al.	Non-NAFLD<br>NAFLD	15 (79.0%)<br>11 (69.0%)	45.2±10.4<br>53.1±7.5	46.7±6.7<br>50.2±5.4	23.8±13.0 |29.9±43.4<br>23.8±12.0 |26.0±28.0	***0.1±0.3<br>*** 0.7±0.9	-	-
Aron-Wisnewsky et al.	Mild OSA<br>Moderate OSA<br>Severe OSA	Not presented	43.5±9.7<br>41.4±11.5<br>48.4±11.3	45.7±5.7<br>46.6±6.8<br>48.3±7.0	21.7±7.8|30.4±19.3<br>22.9±8.4|29.7±20.9<br>23.3±6.6|33.6±16.3	30 (93.9%)<br>28 (82.4%)<br>28 (82.4%)	2 (6.1%)<br>4 (11.8%)<br>6 (17.7%)	0 (0.0%)<br>2 (5.9%)<br>0 (0.0%)
Fu et al.	No OSA<br>Mild OSA<br>Moderate/ Severe OSA	§ 94 (70.1%)<br>§§ 23 (46.9%)	§30.9±0.8<br>§§31.7±1.4	§41.1±0.5<br>§§ 41.8±0.9	31.7±3.1|49.2±4.3<br>38.4±3.8|64.3±7.0<br>46.9±4.1|84.0±9.0	18 (20.0%)<br>10 (21.3%)<br>4 (8.7%)	64 (71.1%)<br>33 (70.2%)<br>29 (63.0%)	8 (8.9%)<br>4 (8.5%)<br>13 (28.3%)
Corey et al.	Non-NAFLD<br>NAFLD	45 (91.8%)<br>87 (79.1%)	40.3±10.4<br>47.8±10.4	45.7±5.5<br>47.5±8.6	- | 16.9±7.9<br>- | 28.3±27.1	-<br>32 (29.1%)	-<br>57 (51.8%)	-<br>21 (19.1%)

*** ballooning score (not classified in stages)

2.9 Cohort studies Ballooning

Author<br>n= (%)	Outcome definition	Gender Female	Age	BMI	AST|ALT	Ballooning stage 0	Ballooning stage 1	Ballooning stage 2
Polotsky et al.	Mild OSA<br>Moderate/severe OSA	42 (93.3%)<br>33 (73.3%)	38.8±8.1<br>43.3±10.2	48.1±7.9<br>49.9±7.9	17.5±6.6 | 11.1±6.1<br>17.8±5.9 | 12.3±6.2	Not presented<br>Only classified in figure	-	-
Weingarten et al.	No/mild OSA<br><br>Moderate/severe OSA	106 (84.8%)<br><br>63 (67.7%)	47.7±11.1<br><br>48.9±10.4	47.0±7.6<br><br>49.2±10.8	28.0±11.0|36.0±24<br><br>28.0±13.0|38.0±19.0	Not presented	-	-
Kallwitz et al.	Mild OSA<br>Moderate/severe OSA	37 (87.0%)<br>26 (60.0%)	42.8±12.1<br>44.5±9.7	51.7±8.5<br>57.2±12.6	*26±15| 35±27	*42 (49.0%)	*31 (37%)	*11 (13.0%)

* In general, not specific to a group

2.10 Cross-sectional studies Lobular inflammation

**Author n= (%)**	**Outcome definition**	**Gender Female**	**Age**	**BMI**	**AST|ALT**	**Lobular inflammation stage 0**	**Lobular inflammation stage 1**	**Lobular inflammation stage 2**
Aron-Wisnewsky et al.	Mild OSA<br>Moderate OSA<br>Severe OSA	Not presented	43.5±9.7<br>41.4±11.5<br>48.4±11.3	45.7±5.7<br>46.6±6.8<br>48.3±7.0	21.7±7.8|30.4±19.3<br>22.9±8.4|29.7±20.9<br>23.3±6.6|33.6±16.3	23 (69.7%)<br>17 (50.0%)<br>14 (41.2%)	9 (27.3%)<br>15 (44.1%)<br>19 (55.9%)	1 (3.0%)<br>2 (5.9%)<br>1 (2.9%)
Fu et al.	No OSA<br>Mild OSA<br>Moderate/<br>Severe OSA	§ 94 (70.1%)<br>§§ 23 (46.9%)	§30.9±0.8<br>§§31.7±1.4	§41.1±0.5<br>§§ 41.8±0.9	31.7±3.1|49.2±4.3<br>38.4±3.8|64.3±7.0<br>46.9±4.1|84.0±9.0	38 (42.2%)<br>13 (27.7%)<br>7 (15.2%)	39 (43.3%)<br>29 (61.7%)<br>27 (58.7%)	13 (14.5%)<br>5 (10.6%)<br>9 (26.1%)
Corey et al.	Non-NAFLD<br>NAFLD	45 (91.8%)<br>87 (79.1%)	40.3±10.4<br>47.8±10.4	45.7±5.5<br>47.5±8.6	- | 16.9±7.9<br>- | 28.3±27.1	-<br>34 (30.9%)	-<br>58 (52.7%)	-<br>18 (16.4%)

2.10 Cohort studies Lobular inflammation

**Author n= (%)**	**Outcome definition**	**Gender Female**	**Age**	**BMI**	**AST|ALT**	**Lobular inflammation stage 0**	**Lobular inflammation stage 1**	**Lobular inflammation stage 2**
Polotsky et al.	Mild OSA<br>Moderate/severe OSA	42 (93.3%)<br>33 (73.3%)	38.8±8.1<br>43.3±10.2	48.1±7.9<br>49.9±7.9	17.5±6.6 | 11.1±6.1<br>17.8±5.9 | 12.3±6.2	Not presented<br>Only classified in figure	-	-

----

2.11 Cross-sectional studies Fibrosis

Author n= (%)	Outcome definition	Gender Female	Age	BMI	AST|ALT	Fibrosis stage 0	Fibrosis stage 1	Fibrosis stage 2	Fibrosis stage 3
Jouët et al.	No OSA<br>Moderate OSA<br>Severe OSA	8 (88.9%)<br>32 (86.5%)<br>12 (92.3%)	30.9±11.5<br>38.2±11.3<br>44.5±5.9	45.5±5.5<br>46.7±7.4<br>54.6±9.8	** 23±15 | 45±18	** 0 (0.0%)	** 29 (46.8%)	** 2 (3.2%)	** 2 (3.2%)
Aron-Wisnewsky et al.	Mild OSA<br>Moderate OSA<br>Severe OSA	Not presented	43.5±9.7<br>41.4±11.5<br>48.4±11.3	45.7±5.7<br>46.6±6.8<br>48.3±7.0	21.7±7.8|30.4±19.3<br>22.9±8.4|29.7±20.9<br>23.3±6.6|33.6±16.3	15 (45.5%)<br>9 (29.4%)<br>5 (14.7%)	13 (39.4%)<br>15 (44.1%)<br>15 (44.1%)	4 (12.1%)<br>8 (23.5%)<br>14 (41.2%)	1 (3.0%)<br>1 (2.9%)<br>0 (0.0%)
Fu et al.	No OSA<br>Mild OSA<br>Moderate/ Severe OSA	§ 94 (70.1%)<br>§§ 23 (46.9%)	§30.9±0.8<br>§§31.7±1.4	§41.1±0.5<br>§§ 41.8±0.9	31.7±3.1|49.2±4.3<br>38.4±3.8|64.3±7.0<br>46.9±4.1|84.0±9.0	38 (42.2)<br>23 (48.9)<br>16 (34.8)	46 (51.1%)<br>23 (48.9%)<br>23 (50.0%)	6 (6.7%)<br>1 (2.2%)<br>6 (13.0%)	0 (0.0%)<br>0 (0.0%)<br>1 (2.2%)
Corey et al.	Non-NAFLD<br>NAFLD	45 (91.8%)<br>87 (79.1%)	40.3±10.4<br>47.8±10.4	45.7±5.5<br>47.5±8.6	- | 16.9±7.9<br>- | 28.3±27.1	-<br>52 (47.3%)	-<br>46 (41.8%)	-<br>8 (7.3%)	-<br>1 (0.9%)

** In general, not specific to a group

2.11 Cohort studies Fibrosis

Author n= (%)	Outcome definition	Gender Female	Age	BMI	AST|ALT	Fibrosis stage 0	Fibrosis stage 1	Fibrosis stage 2	Fibrosis stage 3
Polotsky et al.	Mild OSA<br>Moderate/severe OSA	42 (93.3%)<br>33 (73.3%)	38.8±8.1<br>43.3±10.2	48.1±7.9<br>49.9±7.9	17.5±6.6 | 11.1±6.1<br>17.8±5.9 | 12.3±6.2	Not presented Only classified in figure	-	-	-
Krolow et al.	No/mild OSA<br>Moderate/severe OSA	21 (51.2%)<br>5 (50.0%)	57.0±8.2<br>58.7±7.0	*<br>33.0±5.3	& 4 (9.8%) | 17 (41.5%)<br>& 2 (20.0%) | 3 (30.0%)	0 (0.0%)<br>0 (0.0%)	10 (33.3%)<br>5 (50.0%)	20 (66.7%)<br>5 (50.0%)	-
Weingarten et al.	No/mild OSA	106 (84.8%)	47.7±11.1<br><br>48.9±10.4	47.0±7.6<br><br>49.2±10.8	28.0±11.0|36.0±24<br><br>28.0±13.0|38.0±19.0	Not presented	-	-	-

	Moderate/severe OSA	63 (67.7%)							
Kallwitz et al.	Mild OSA<br>Moderate/severe OSA	37 (87.0%)<br>26 (60.0%)	42.8±12.1<br>44.5±9.7	51.7±8.5<br>57.2±12.6	*26±15| 35±27	** 69 (81.0%)	** 7 (8.0%)	** 5 (6.0%)	** 3 (4.0%)

** In general, not specific to a group

2.12 Cross-sectional studies Fibro inflammation

Author n= (%)	Outcome definition	Gender Female	Age	BMI	AST|ALT	Fibro inflammation Absent	Fibro inflammation Moderate	Fibro inflammation Severe
Aron-Wisnewsky et al.	Mild OSA<br>Moderate OSA<br>Severe OSA	Not presented	43.5±9.7<br>41.4±11.5<br>48.4±11.3	45.7±5.7<br>46.6±6.8<br>48.3±7.0	21.7±7.8|30.4±19.3<br>22.9±8.4|29.7±20.9<br>23.3±6.6|33.6±16.3	9 (27.3%)<br>18 (54.6%)<br>6 (18.0%)	7 (20.6%)<br>14 (41.2%)<br>13 (38.2%)	3 (8.8%)<br>14 (41.2%)<br>17 (50.0%)

2.12 Cohort studies Fibro inflammation

Author n= (%)	Outcome definition	Gender Female	Age	BMI	AST|ALT	Fibro inflammation Absent	Fibro inflammation Moderate	Fibro inflammation Severe
Agrawal et al.	NAFLD<br>OSA present	38 (38.0%)<br>5 (22.0%)	41.0±11.0<br>46.0±12.0	28.3±4.4<br>32.2±7.6	45 (33-66) | 71 (44-96)<br>26 (24-44) | 41 (27-64)	0 (0.0%)<br>0 (0.0%)	19 (22.0%)<br>8 (9.0%)	6 (35.0%)<br>4 (24.0%)

2.13 Cross-sectional studies Liver volume cm^3 or Liver/Kidney echo ratio

Author	Outcome definition	Gender Female	Age	BMI	AST|ALT	Liver volume cm^3	Liver/Kidney echo ratio
Scartabelli et al.	No OSA<br>With OSA	27 (100.0%)<br>70 (100.0%)	40.8±9.5<br>48.8±10.3	48.5±6.0<br>50.0±8.1	22.1±13.4 | 27.0±±14.1<br>24.4±12.7 | 30.9±17.8	344.6±144.5<br>487.9±261.3	-
Turkay et al.	No OSA<br>Mild OSA<br>Moderate OSA<br>Severe OSA	§ 9 (25.7%)<br>* 18 (24.4%)	§47.2±14.4<br>*51.4±12.4	§29.2±5.2<br>*33.1±7.4	18 (12-26) | 23.5(11-52)<br>19 (11-51) | 25 (12-144)<br>21 (12-56) | 26 (10-75)<br>18 (11-52) | 22 (5-74)	-	§§Non NAFLD<br>Mild NAFLD<br>Moderate NAFLD<br>Severe NAFLD
Zhang, L et al.	No OSA<br>Moderate OSA<br>Severe OSA	16 (84.2%)<br>57 (70.4%)<br>21 (39.6%)	27.8±7.3<br>30.2±7.6<br>32.1±7.8	38.7±3.5<br>41.3±5.9<br>45.5±6.8	26.4±14.4 | 44.6±28.9<br>33.9±26.3 | 64.3±52.5<br>41.9±28.5 | 78.8±54.2	-	§§§
Bhatt et al.	With OSA with NAFLD<br>With OSA no NAFLD<br>No OSA with NAFLD<br>No OSA No NAFLD	60 (48.4%)<br>22 (46.8%)<br>18 (41.0%)<br>8 (32.0%)	44.8±9.1<br>44.2±9.1<br>39.5±10.5<br>41.0±8.5	33.3±7.9<br>32.5±14.2<br>31.0±8.3<br>28.5±8.6	44.5±15.9 | 60.9±10.3<br>41.4±22.1 | 54.2±12.9<br>39.6±19.6 | 52.3±11.9<br>31.6± 15.9 | 50.9±10.9	-	§§§

§ Baseline expressed towards non-NAFLD, not towards OSA.
* Baseline expressed towards NAFLD, not towards OSA.
§§ no actual results were presented on the outcome—only a categorical expression of the NAFLD of the echo.

§§§ no actual results were presented on the outcome of the echo.

2.13 Cohort studies Liver volume cm^3 or Liver/Kidney echo ratio

Author	Outcome definition	Gender Female	Age	BMI	AST|ALT	Liver volume	Liver/Kidney echo ratio
Zhang, Y.X et al.	Mild-moderate OSA<br>Severe OSA	41 (51.9%<br>34 (66.3%)	30.0±9.0<br>34.0±10.0	37.2±6.4<br>41.0±6.9	28.5±16.6 | 49.2±42.5<br>39.4±25.8 | 69.0±54.3	-	@0.82 ± 0.27<br>@0.68 ± 0.22<br><br># 60 (75.0%)<br># 96 (96.0%)<br><br>%−2.306 ± 1.351

							%−1.468 ± 1.432

(@LSR liver/spleen Hounsfield unit ratio), (#NAFLD percentage), (% NFS NAFLD fibrosis score)
